# Interventional therapy for a variant central retinal artery occlusion: A case report

**DOI:** 10.1097/MD.0000000000046319

**Published:** 2025-11-28

**Authors:** Kunming Xie, Wei Li, Qingbo Wang

**Affiliations:** a Department of Neurosurgery, Weifang People’s Hospital, Shandong Second Medical University, Weifang, Shandong, China; b Department of Neurosurgery, Weifang Binhai Economic and Technological Development Zone People’s Hospital, Weifang, Shandong, China.

**Keywords:** central retinal artery occlusion, interventional treatment, superselective middle meningeal artery thrombolysis

## Abstract

**Rationale:**

Summarize the clinical experience of interventional thrombolysis treatment for a variant central retinal artery occlusion(CRAO).

**Patient concerns:**

The patient experienced a sudden decline in left eye vision for 3 hours.

**Diagnoses and interventions:**

One case of a special type of CRAO was reported, treated with microcatheter super selective middle meningeal artery thrombolysis.

**Outcomes:**

The blood supply of the central artery on both sides of the patient comes from the branch of the middle meningeal artery. The middle meningeal artery was selected by microcatheter and treated with urokinase via microcatheter. The patient’s vision was significantly improved.

**Lessons:**

Transcatheter super selective ophthalmic artery thrombolysis is currently a favorable surgical approach for treating CRAO. Accumulating clinical experience with variant ophthalmic arteries is beneficial for the prognosis of patients with CRAO.

## 1. Introduction

The majority of the central retinal artery originates from the ophthalmic artery and belongs to the terminal artery with an average diameter of 0.42 mm. It mainly participates in the blood supply to the inner layer of the retina. Acute blockage of the central retinal artery can cause retinal ischemia and hypoxia, leading to sudden blindness in the affected eye.^[1]^ The ophthalmic artery usually originates from the siphon of the internal carotid artery, and there are few patients with ophthalmic artery loss. This article introduces a special case of central retinal artery occlusion (CRAO) with ophthalmic artery loss, aiming to accumulate clinical experience on this disease and improve patient prognosis.

## 2. Case report

Patient male, 46 years old. He came for treatment due to a sudden loss of vision in his left eye for 3 hours. The patient experienced a sudden decline in left eye vision without obvious cause about 3 hours ago, with blurred vision, only light sensation, accompanied by headache, no nausea or vomiting, no consciousness disorders, no mouth or eye deviation, and no limb convulsions. The patient was admitted to our department for “central retinal artery occlusion” in the outpatient department. Medical history: cerebral infarction. Admission physical examination: clear consciousness, good mental state, speech was relevant, left eye vision only had light perception, limbs followed instructions to move, muscle strength and tension were normal.

**Figure 1.**
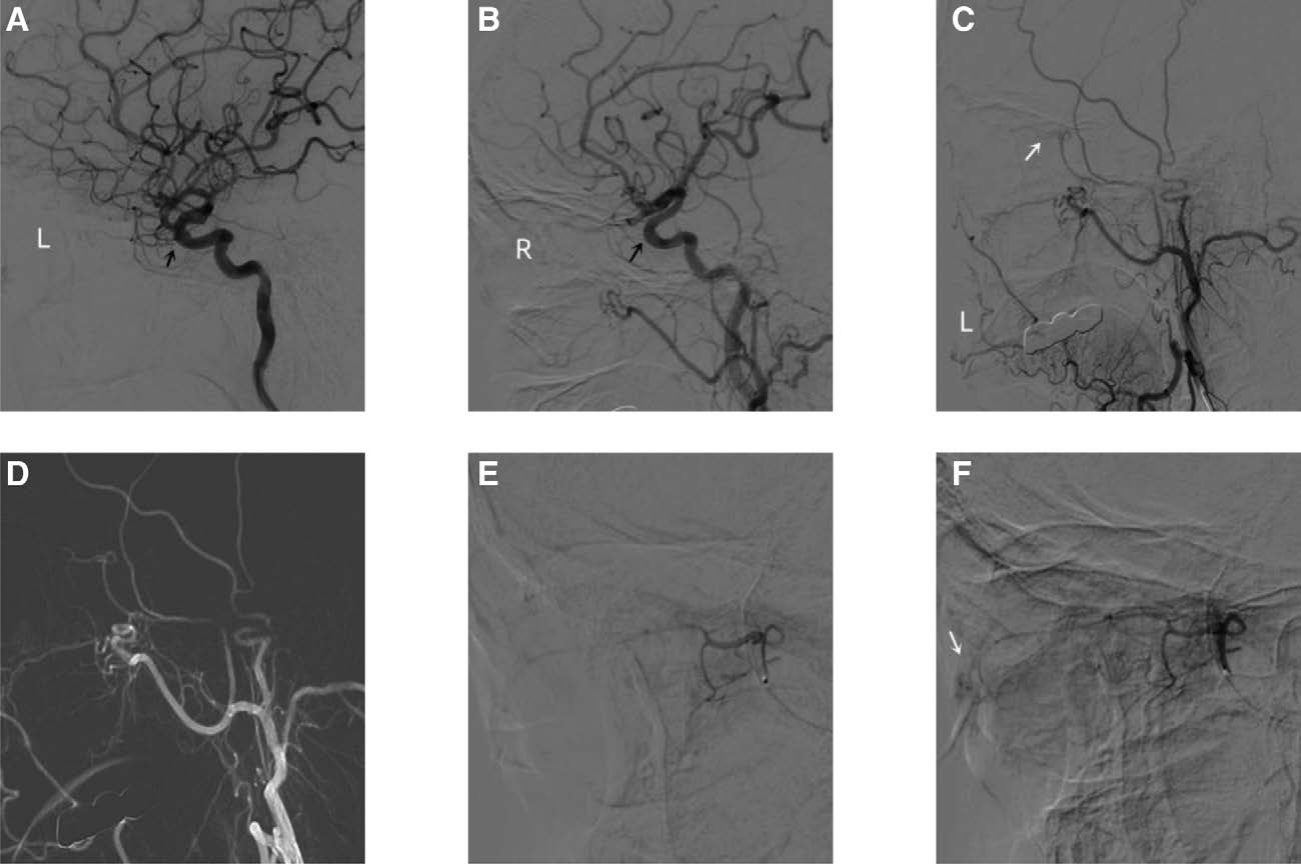
Superselective left middle meningeal artery thrombolysis for central retinal artery occlusion. (A) Left internal carotid artery angiography showed the left ophthalmic artery was occluded (see black arrow). (B) Right common carotid artery angiography showed occlusion of the right ophthalmic artery (see black arrow). (C) Left external carotid artery angiography showed the retinal vascular network supplied by a branch of the left middle meningeal artery, with a faint contrast (middle meningeal artery, see white arrow). (D, E) Under the guidance of a guidewire, a marathon microcatheter was placed at the distal end of the middle meningeal artery branch in the pathway diagram, and urokinase had been slowly injected. (F) Microcapillary angiography showed significant improvement in the visualization of the central retinal artery (see white arrow), and the patient’s vision returned to its pre-disease state.

Surgical procedure (Fig. 1): The patient was placed in the supine position, and the surgical area was routinely disinfected and draped with sterile towels. Local infiltration anesthesia was applied, and Seldinger technique was used to puncture the right femoral artery and insert a 6F arterial sheath. First, the left internal carotid artery angiography was performed, revealing mild stenosis at the origin of the left internal carotid artery, occlusion of the left ophthalmic artery, and no clear retinal blood supply artery. At the same time, a cystic aneurysm with a size of approximately 2.46 mm × 0.92 mm was found in the ophthalmic segment of the left internal carotid artery. Then, the right internal carotid artery angiography was performed, revealing occlusion of the right ophthalmic artery and no clear retinal blood supply artery. Further left external carotid angiography revealed compensatory supply of the retinal vascular network by the left middle meningeal artery branch. Whole body heparinization was performed, and a single curved catheter was placed at the beginning of the left external carotid artery under the guidance of a loach guide wire. In the pathway diagram, a Marathon microcatheter was guided by a Traxcess-14 microcatheter to reach the distal end of the left middle meningeal artery branch. Angiography showed that the microcatheter was in good position, and the retinal vascular network appeared faint. In 7,50,000 units of urokinase were slowly injected through the microcatheter, and the imaging of the retinal arterial network showed improved. During the operation, the patient reported that the left eye vision had recovered from only weak light sensation to several fingers in front of the eye, improved to the pre-onset visual state gradually. Intraoperative CT scan did not show any signs of intracranial hemorrhage. Then the operation was ended.

## 3. Discussion

CRAO is an acute blinding disease, with a rapid onset^[1]^ and an incidence rate of 0.01% to 0.05%. Because the retina is very sensitive to ischemia and hypoxia, if not treated in time, it will cause irreversible blindness.^[2]^ The central retinal artery belongs to the terminal artery of the ophthalmic artery, with a small diameter and no anastomotic branches. Once it is occluded, it is difficult for drugs to quickly reach the lesion site with sufficient drug concentration through systemic medication, resulting in poor treatment efficacy. The recovery of retinal artery blood flow is the key to curing CRAO, and currently, the treatment of CRAO with selective ophthalmic artery urokinase thrombolysis via microcatheter has achieved good prognosis.^[3]^ Ziyal^[4]^ pointed out that the ophthalmic artery normally originates from the internal carotid artery. However, variations in the ophthalmic artery are common in clinical practice. Gregg^[5]^ reported 1 case where the ophthalmic artery originated from the posterior communicating artery and 1 case from the anterior cerebral artery; Sade^[6]^ found 1 case where the left ophthalmic artery originated from the main trunk of the basilar artery. The ophthalmic artery often has communicating branches with the middle meningeal artery, but it is rare for the middle meningeal artery branch to completely eliminate the ophthalmic artery, and even rarer for both sides to originate from the middle meningeal artery.

This study treated a CRAO patient with ophthalmic artery variation in clinical practice, where both ophthalmic arteries originate from the middle meningeal artery (Fig. 1). The patient’s left eye was blind. During the treatment of this case, it was found that the left ophthalmic artery was not visible, and it was initially thought to be a left ophthalmic artery occlusion. However, the inner wall of the internal carotid artery in the segment of the ophthalmic artery was smooth, and the selection of the ophthalmic artery failed. Another right internal carotid artery angiography was performed, and it was found that the right ophthalmic artery was also not visible, but the right eye had normal vision. Therefore, it was considered that the retinal blood supply source of the patient may not be the ophthalmic artery or ophthalmic artery variation. A left external carotid artery angiography was performed to confirm that the retinal blood supply artery was the middle meningeal artery. A Marathon microcatheter was used to select the left middle meningeal artery branch, and urokinase. The aneurysm in the ophthalmic segment of the left internal carotid artery was regular in shape and small in size, so it was not treated temporarily. Although the treatment process of this case was tortuous, it has accumulated valuable experience for us to treat CRAO in the future.

Some studies have shown that CRAO occurs mostly in the elderly with hypertension,^[7]^ diabetes, atrial fibrillation and carotid atherosclerosis. Retinal ischemia time is the most important influencing factor to determine visual acuity recovery.^[8]^ Thrombolysis time is closely related to visual acuity prognosis.^[9,10]^ Unfortunately, the onset time of domestic patients is >6 hours. Transcatheter selective ophthalmic artery thrombolysis for CRAO has achieved significant therapeutic effects, and studies have shown that its common complication is cerebral hemorrhage, with an incidence rate of 4%.^[11]^ Therefore, preoperative risk assessment should be improved, such as cerebral vascular malformation, history of cerebral hemorrhage, thrombocytopenia, etc, to reduce surgical complications.

Familiarity with the variations of the central retinal artery and ophthalmic artery is beneficial for the treatment of patients with sudden blindness caused by various types of CRAO. This type of mutation is also of great significance for neurosurgical craniotomy surgery. In standard frontotemporal craniotomy surgery, if patients experience ipsilateral blindness after surgery, the possibility of the ophthalmic artery originating from the middle meningeal artery should be considered.

In summary, superselective ophthalmic artery thrombolysis is currently a favorable surgical approach for treating CRAO. Accumulating clinical experience in ophthalmic artery variation is beneficial for the prognosis of patients with CRAO.

## Author contributions

**Writing – original draft:** Kunming Xie, Wei Li.

**Writing – review & editing:** Wei Li, Qingbo Wang.
